# Quality Assessment of a Manufactured Bell Using a 3D Scanning Process

**DOI:** 10.3390/s20247057

**Published:** 2020-12-09

**Authors:** Dawid Cekus, Paweł Kwiatoń, Maciej Nadolski, Krzysztof Sokół

**Affiliations:** 1 Department of Mechanics and Machine Design Fundamentals, Faculty of Mechanical Engineering and Computer Science, Czestochowa University of Technology, ul. Dąbrowskiego 73, 42-201 Czestochowa, Poland; kwiaton@imipkm.pcz.pl (P.K.); sokol@imipkm.pcz.pl (K.S.); 2 Department of Metallurgy and Metal Technology, Faculty of Production Engineering and Materials Technology, Czestochowa University of Technology, Al. Armii Krajowej 19, 42-201 Czestochowa, Poland; maciej.nadolski@pcz.pl

**Keywords:** laser scanning, cast inspection, sensor technology and application, point cloud, acoustics, frequency analysis

## Abstract

Bells as percussion instruments have been known to humanity for ages. The casting process, the shape and the materials have changed over the years. The functional properties of bells depend on the casting quality and the generated sound. The casting quality is related to the shape, material and technology. The acoustic quality must fulfill specific parameters. This work concerns the assessment of the quality of the manufactured bells and the possibility of replacing tin bronze with a material with similar mechanical properties. Therefore, bell bronze—which is dedicated to this type of work—and aluminum bronze—which is characterized by high strength and hardness, have been applied to casting. The laser scanning technique was used to assess the quality of castings. Based on a point cloud, an optimized mesh was generated from which the 3D model was finally created. On the basis of the CAD model, the defects resulting from the casting process were determined in the form of graphical comparison. The correctness of the selected foundry shrinkage of the selected material was also determined. The manufactured bells were also assessed for sound quality. Vibration frequencies were determined using a vibration analyzer and free software Wavanal. The experimental results were compared with the ones obtained from the numerical frequency analysis. With the help of tests, the quality of the bells was assessed, and it was determined whether aluminum bronze meets the acoustic criteria. The presented method can be used in the development of bell templates. These templates will allow the bell to obtain the correct shape and acoustic quality without the need for a tuning process.

## 1. Introduction

Bells are an example of musical instruments called idiophones. They have been known to mankind since ancient times, and the first of them was made in China in 3000 BC [1]. In the past, bells were made of copper or iron sheets. Currently, the basic materials from which the bells are made are copper–tin alloys, so-called bell bronze [2,3]. However, bell bronze is a structurally complex material and its parameters depend on many properties and technological aspects of the casting process [4,5]. The manufactured bell should be subjected to detailed qualitative analysis. The accuracy of the bell cast is verified by the acoustic properties (frequency analysis). Often, material tests are also carried out, including the research on the structure of the casting and its porosity. Thanks to technological developments, 3D scanning is a new method that allows one to determine the quality of the manufactured bell. It is possible to compare the designed CAD model with the scanned one. However, this method has some disadvantages. The scanning process is laborious and time-consuming and the efficiency depends mainly on the skills of the operator [6]. The laser scanning process is also unable to detect internal defects of castings.

Laser scanning technology is currently one of the most common methods used in the research of manufactured parts. It is an indispensable part of the reverse engineering (RE) process that allows one to obtain a CAD model in the case of lack of proper technical documentation [7,8]. The main objective of the 3D scanning process is the most accurate representation of a real object in a digital form [9]. The 3D scanning technology uses an intense light source with a detector which cooperates with the software to recreate the scanned object [10]. The obtained 3D model can be used to reproduce the technical documentation, perform a strength analysis or determine the quality of the manufactured product without destructive methods. The development of this technology has made it popular in areas such as automotive industry [8,9,10], medicine [11], archaeology [12], arts [13], cultural heritage reconstruction [14], casting [15] and forging [16]. The paper [8] presents studies concerning the application of the 3D scanning technology to sheet-metal parts and sets of automotive dies. A detailed description of the scanning process was presented, which consisted of sensor calibration, preparation of scanned parts, receiving scanning data and post-processing. The use of a 3D scanner to inspect the body of a motorcycle carburetor is presented in [9]. The scanning methodology and measurement sequences were discussed. Finally, a 3D model was obtained, on the basis of which the authors developed a construction drawing. Babić et al. [11] introduced the laser scanning method to determine the fatigue life assessment of a prosthesis. With the help of the obtained point cloud, a prosthesis was modeled, which was subjected to numerical analysis using the finite element method (FEM). Karasik and Smilansky [12] presented the usage of a laser scanner during archaeological research on pottery. In [12], a positioning algorithm was also developed that allows one to determine the axes of symmetry of the analyzed ceramic objects. The reconstruction process of the sculpture by means of 3D scanning was presented in [13], where the reverse distance method was used to repair the point cloud and restore the surface. The usage of laser scanning for the reconstruction of an ancient musical instrument is presented in [14]. Based on the scanned fragments of the shell, the musical instrument was manufactured using modern methods. The reconstructed lyre was subjected to acoustic tests in which its spectral centroid and skewness and the standard deviations were determined. In [15] the research on the improvement of the aluminum die casting mold with the use of a laser scanning tool was presented. The differences between the designed model and the cast object were shown. The problem with correction vectors was also discussed. A new measurement method based on non-contact 3D scanning techniques and the analysis of geometric changes in the die forging process has been described in [16]. In addition, indirect scanning was presented. The errors that arose during the measurements as a result of changing the reflection angle were determined.

Several methods of determining the correctness of the obtained cast can be found in the literature. Scanning electron microscope tests are most often used to define the appropriate structural composition [17,18]. Among non-destructive tests, in addition to the standard approach, with the use of the coordinate measuring machine (CMM), laser scanning is being increasingly used. The quality evaluation of the elements manufactured with the application of a 3D printer is presented in [19]. The 3D-SLSS measurement method was used during the digitization of the clay object. Particular attention was paid to the phenomenon of distortion, which is common in additive manufacturing. Lazarevic et al. [20] presented a dimensional inspection of the object based on two methods: CMM and 3D scanning. The inspection was performed on a plate milled by two strategies: up-milling and down-milling. It has been shown that the digitization process can be considered as an appropriate tool for evaluation of geometric deviations. In the case of bells, the 3D scanning process is most often used for reconstruction and further numerical analysis. In [21] the authors presented the use of reverse engineering in the research on a bell’s acoustics. The scanned bell was divided into finite elements and frequency analysis was performed. Five vibration frequencies (partials) were determined and experimental verification was performed.

In addition to the dimensions and structure of a casting, the sound is the most important criterion for assessing the quality of a bell. According to the theory of tuned bells, five partials have the greatest influences on the listener’s perception of sound [22]. Debut et al. [23] presented a microstructural analysis of a medieval bell. Characteristic material data such as Young’s modulus and density were determined. A frequency analysis was performed, on the basis of which the five frequencies were obtained. Moreover, the bell velocity power spectrum was also shown. The acoustic research on the cathedral bell was described in [24]. The presented methodology took into account the mass and stiffness matrices and the displacement vector. The obtained frequency results were compared with the data of other bells described in the literature. The acoustic tests of the bell involving the extraction of the beating characteristics and damping coefficients are described in [25]. The model assumes that the bell can be represented by a combination of an asymmetric ring and a cylindrical shell. The computational model was developed on the basis of continuous wavelet transform (CWT), Shannon entropy cost function and mother wavelet. Gołaś [26] presented a mathematical model of sound synthesis based on Laplace transforms and the Galerkin method. The developed method of sound synthesis was used to determine the natural surface vibrations and the propagation of sound in its surroundings. The obtained structural parameters of the sound during the synthesis can be used at the stage of designing musical instruments. Modal synthesis using the Faust physical model and the finite element method is presented in [27]. A frequency analysis was performed, on the basis of which the results of the characteristic bell partials were obtained. The possibilities of open-source software for modeling and dynamic analysis of acoustic instruments were also shown. Lee et al. [28] presented a technique for determining bell beat maps based on the operational deflection shape (ODS) method. MATLAB software was used to process the results. The moment at which the bell produces clear beats was specified. An innovative, non-destructive method of bell tuning is presented in [29]. The technique is a combination of optimization methods, dynamic modeling and modifying structured instruments. Material tests of bells made of aluminum bronze are presented in [30]. Isochronous aging of the obtained metal alloys and their acoustic properties were also analyzed.

Apart from the frequency analysis, in the literature one can also find works on the analytical approach to bell dynamics [31,32,33]. In the article [31], a dynamic model of the church bell was developed, depending on the Lagrange equations and the model of concentrated parameters. The phenomenon of contact during hitting a bell was described. The obtained results were verified based on the available experimental results, and the risk of damage to the system was indicated. The mathematical model of the church bell, taking into account the new type of bifurcation, is presented in [32]. Typical bifurcation scenarios and the impact of the yoke geometry on the system dynamics were investigated. The change in stability and its individual ranges were determined. A mathematical approach using nonlinear ordinary differential equations during bell dynamic analysis was included in [33]. The developed algorithm enables the analysis of free, damped and undamped oscillations. In addition, the evaluation of damping equivalence and resonance inducing functions was done.

**Figure 1:**
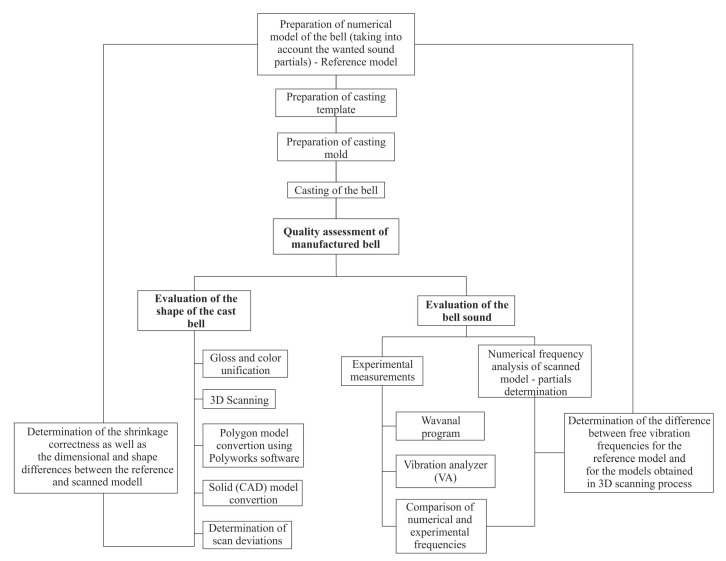
Flowchart of the bell quality assessment.

This article deals with the general analysis of the quality of the produced bells (Figure 1). In order to conduct comparative tests, two castings were made, similar in terms of shape and mold technology. The castings differed only in the material used: bell bronze—CuSn20 (chemical composition: 80% copper, 20% tin) and aluminum bronze—CuAl10Fe3Mn2 (chemical composition: 85% copper, 10% aluminum, 3% iron, 2% manganese). Aluminum bronze was chosen because materials that can replace high tin bronzes (obtaining tin in the coming years will be more difficult and more expensive) are desirable. In Europe, Morris Stirling made a cast iron bell in 1610 and Jacob Maier made a steel bell in 1851. Maréchal refers in his work [34] to German and American literature on making bells of glass, porcelain and aluminum, on the basis of which one can state that the bells made of aluminum were characterized by “pleasant to ear tone and long resonance”. The quality assessment was divided into two stages. The first part concerned the correctness of the obtained casting. In this step, the bell was scanned using a measuring arm equipped with a laser scanning head. Using the SolidWorks and Polyworks software, the obtained scans were compared with the designed CAD model. For the second stage, acoustic tests of the received bells are presented. Basic acoustic properties that allow for defining the correctness of the assumed criteria were determined. The aim is to develop a casting technology in which sounds (vibration frequencies—partials) of the manufactured bell will not differ significantly from the numerical model. This approach will minimize the need to carry out the process of tuning. The novelty of the work is the verification of the dimensions of the template (CAD model) with the casting, which allows one to determine the phenomenon of inhibited and free shrinkage in the construction of bells.

## 2. Quality Assessment of the Bell Casting Process

The quality of the obtained castings was assessed using the 3D scanning process. This process, in addition to the identification of the casting surface errors, was also used to verify the correctness of the introduced material shrinkage. Material shrinkage (foundry shrinkage) is a change in material dimensions during solidification or cooling process. Two bells differing in material (Table 1) were analyzed. The first one was made of bell bronze (CuSn20), which is the most commonly used alloy in the production of these instruments. The second material was aluminum bronze (CuAl10Fe3Mn2), which was proposed as an alternative to traditional materials. It has similar acoustic and material properties. The material’s shrinkage was adopted according to the traditional approach to bell casting. In the casting mold, a scale factor of 1.011 was assumed only in the horizontal direction.

### 2.1. Research Methods

**Figure 2:**
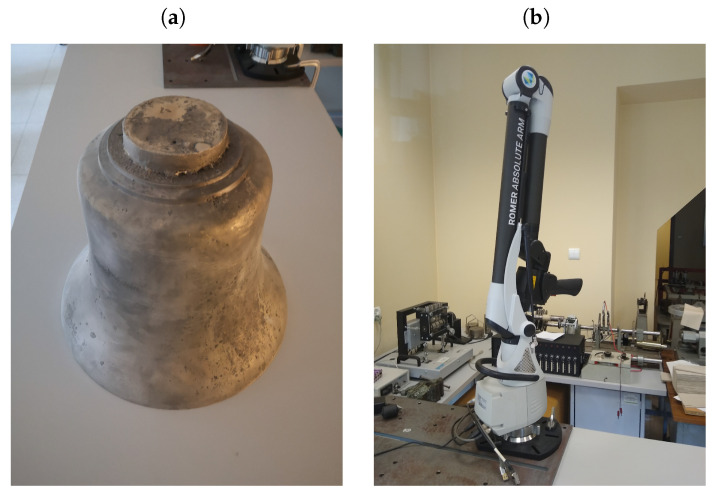
(**a**) CuSn20 bell during uniform gloss and color of the surface; (**b**) ROMER Absolute ARM.

The first operation prior to the scanning process is to apply a matting powder to the bell to unify the gloss and color of the scanned surface (Figure 2a). Then, using the Hexagon [35] ROMER Absolute Arm (Figure 2b) equipped with a laser scanning head RS3 (Table 2), the measurements of the bells were done, on the basis of which clouds were obtained.

**Figure 3:**
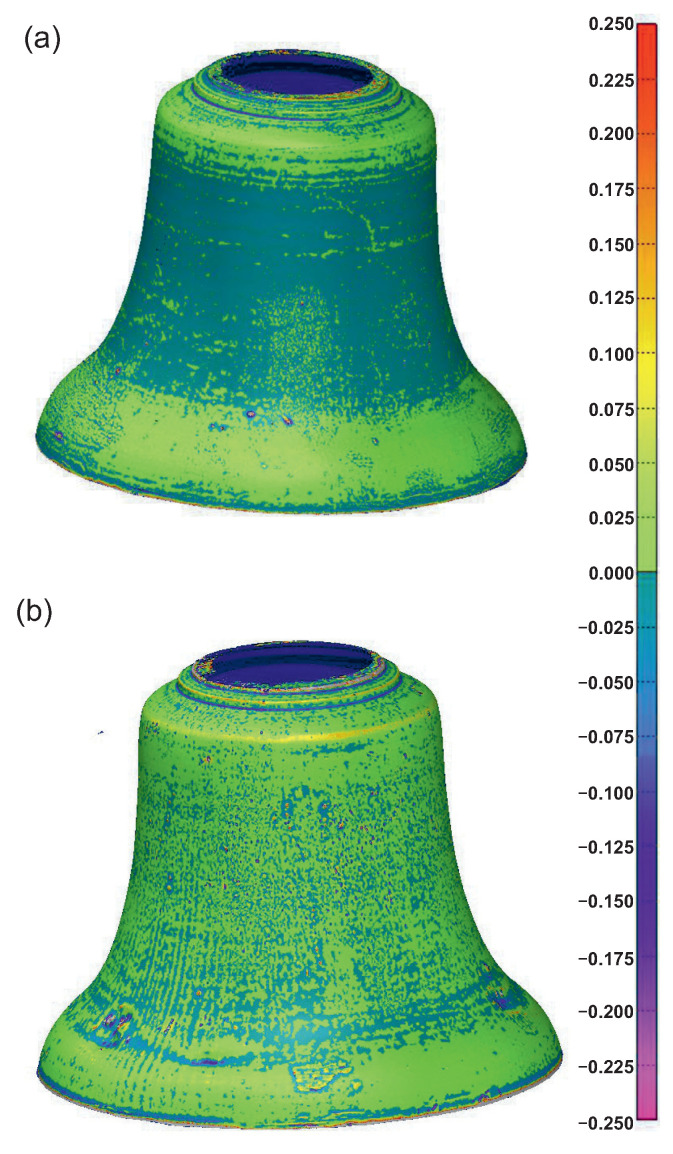
Comparison of the deviations (*mm* unit) of the created meshes: (**a**) CuSn20; (**b**) CuAl10Fe3Mn2.

In the next stage, the point clouds were rebuilt into a polygon model. Finally, a CAD model of the scanned bell was made in SolidWorks software [36]. The prepared model of the cast bell and its CAD model were imported to the Polyworks Inspector [37] in order to determine the deviations of the casting shape. Based on the position markers, both models were set in the appropriate positions relative to each other, and then the maps depicted in Figure 3 were drawn.

The scope of individual tasks during the quality assessment included:

- Uniform gloss and color of the scanned surface;
- 3D scanning—creation of a point cloud;
- Conversion to a polygon model;
- Conversion to a solid model (CAD);
- Determination of the deviations;
- Comparison of the scans received with the assumed project (prototype/reference model);
- Results analysis.

### 2.2. Results

**Figure 4:**
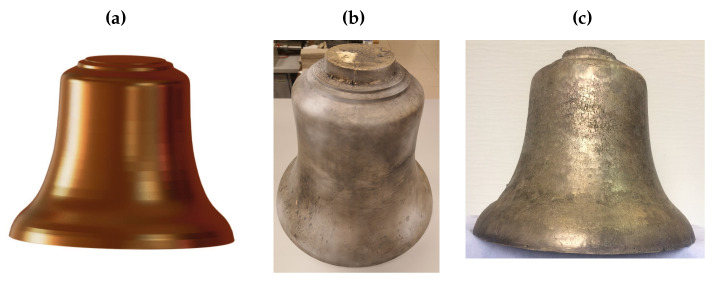
Analyzed bells: (**a**) CAD model; (**b**) CuSn20; (**c**) CuAl10Fe3Mn2.

**Figure 5:**
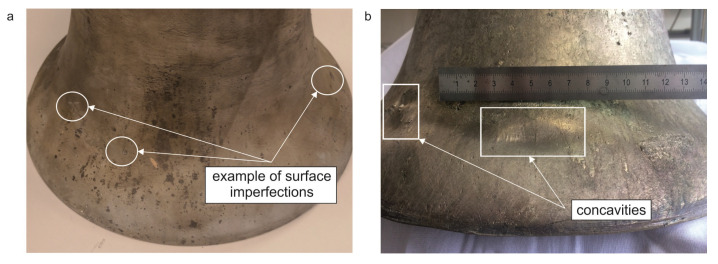
Visual comparison of analyzed bells (**a**) CuSn20; (**b**) CuAl10Fe3Mn2.

The quality of scans was assessed using the “Body Compare” command in SolidWorks software. The first assessment of the accuracy of the obtained casting was a visual comparison (Figure 4). Figure 5 shows minor surface imperfections for a bell made of CuSn20 material (Figure 4b). The bell made of aluminum bronze (Figure 4c) had greater inaccuracies in the surface obtained.

The Body Compare feature allows one to correlate two groups of objects (CAD models, meshes or scans) to find their differences [38]. Therefore, it was necessary to properly locate both elements. For this purpose, the characteristic points of the scan enabling the reconstruction of its rotation axis were defined. Thus, the models were placed concentrically.

**Figure 6:**
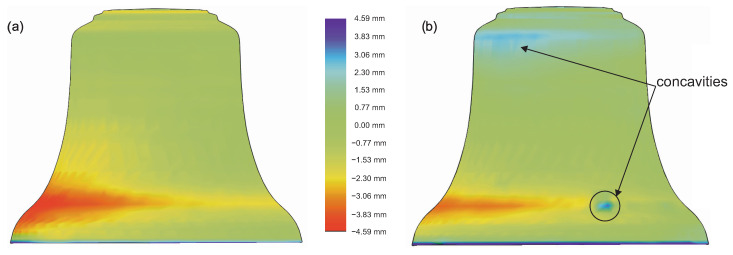
Quality control of the obtained 3D scans—external view: (**a**) CuSn20; (**b**) CuAl10Fe3Mn2.

**Figure 7:**
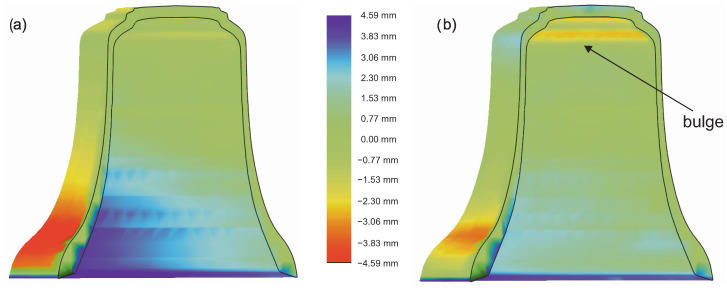
Quality control of the obtained 3D scans—sectional view: (**a**) CuSn20; (**b**) CuAl10Fe3Mn2.

**Figure 8:**
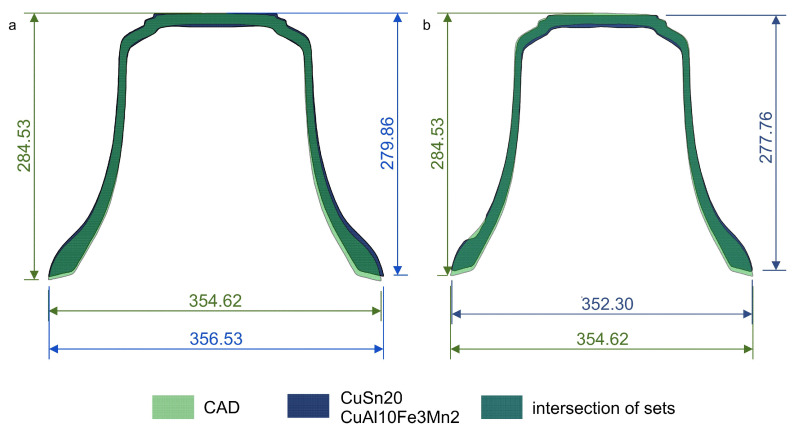
Assessment of the correctness of the selected shrinkage: (**a**) CuSn20; (**b**) CuAl10Fe3Mn2.

The results of the comparative analysis are presented in Figure 6, Figure 7 and Figure 8. On the basis of the obtained results, it can be concluded that cast bells differed from the designed prototype. In both cases, excess material was visible on the outer side (Figure 6). The maximum deviation was 4.59 mm for the casting made of bell bronze, and it was −4.20 mm for the second material. The deviations are related to the mold structure, and more precisely to the process of applying the protective coating. This is one of the disadvantages of so-called hand-made manufacturing, which is not automated. The protective coating and the construction of the entire structure of the mold are performed by human hands. An important aspect in this case is the bell foundry experience.

The assessment criterion, in this case, is that the obtained casting should not have concavities, which will significantly affect its acoustic properties. Such concavities were visible in the bell made of CuAl10Fe3Mn2 material. It can therefore be concluded that the obtained partials differed from those expected. In Figure 7, one can notice the opposite phenomenon on the internal side of the bell. Material losses are visible on the inside compared to the CAD design. In the CuAl10Fe3Mn2 bell (Figure 7b), a bulge in the upper part of the bell was visible. Based on the obtained results, it can be concluded that the mold construction process should be analyzed and improved in a way to minimize the differences in the material. Figure 8 also clearly states that the foundry shrinkage, especially in the vertical direction, should be clarified. The vertical dimensions of the received bells were shorter than in the CAD project by 4.67 mm for bell bronze and by 6.77 mm for aluminum bronze. The horizontal dimensions differed by 1.91 mm (CuSn20) and 2.32 mm (CuAl10Fe3Mn2).

The obtained results of the casting shrinkage tests revealed differences in the dimensions of the diameters of the mold cavities in the considered cases. They resulted from human error, which arose when the dimensions of the template location in relation to the axis of rotation were checked. Based on the measurements, the inhibited shrinkage for high tin bronze is approximately 0.54% and for aluminum bronze 0.62%; free shrinkage is 1.63% or 2.38% respectively. The greater shrinkage of the aluminum bronze is consistent with the literature data. The change in the dimensions of the casting affects the obtained results of the analysis of the components of the bell sound.

## 3. Evaluation of the Bell Sound

The most important step in the quality assessment of the bell casting is the evaluation of its acoustic properties (sound assessment). The sound emitted from a bell is composed by the sum of its specific partials and from residual noise. A partial is a certain frequency from the sound spectrum, given by a certain mode of bell vibration. In the case of bells, which belong to the percussion class of instruments, the partials represent fractional multiples of the fundamental frequency. The sound spectrum of a bell, i.e., the amplitude or intensity of sound versus frequency, is composed of peaks corresponding to the partials, with background given by the bell’s noise. The partials with frequencies bigger than that of the fundamental component of a certain complex sound are known as overtones or upper partials, and they have specific names, in increasing order of their frequencies: hum, prime, tierce, quint, nominal and superquint [39,40,41,42]. The theoretical intervals given in cents relative to the nominal for the lowest five partials are shown in Table 3.

### 3.1. Numerical Research

Numerical tests were performed in order to determine the differences in free vibration frequencies between the reference model (which was used to make the template for the casting) and models obtained on the basis of the 3D scanning process (Figure 4). The SolidWorks simulation software [43,44] was used for numerical analysis, performed with the FEM.

**Figure 9:**
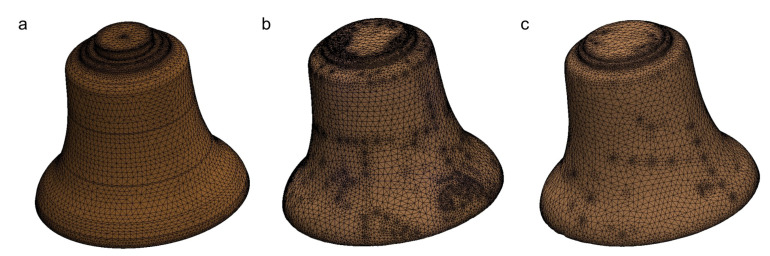
Generated meshes: (**a**) reference model; (**b**) scanned model CuSn20; (**c**) scanned model CuAl10Fe3Mn2.

The reference model did not take into account the linear and volumetric shrinkage that occur for bronze castings and are different for tin bronze and aluminum ones. The template for the casting was prepared in such a way that only the shrinkage that could occur along the circumference was taken into account, while the shape of the rib was not modified along its height. As a result, the height of the cast object was smaller than the reference model, which at the same time affected the volume of the object and the mass. Moreover, the numerical model is idealized and does not have any disadvantages, such as microporosity and shrinkage porosity, which can be found in castings of this type [45,46]. When using FEM, one divides the model into the so-called finite elements. Elements have common points called nodes. The DOF is a node response that is typically described with three translations and three rotations. The DOF number directly depends on the number of finite elements. In the analyzed cases, high quality mesh with parabolic tetrahedral solid elements was used. Assuming the same mesh parameters (including maximum element size = 10 mm and minimum element size = 2 mm) for all tested models, the automatic mesher generates meshes shown in Figure 9.

Figure 9 clearly shows the variety of mesh for each model. The reference model has no imperfections which translate directly into the number of finite elements. However, in the case of scanned models, each imperfection is taken into account during mesh creation. That is clearly visible in Figure 9b when using CuSn20. This feature determines the differences in number of DOF (Table 4).

**Figure 10:**
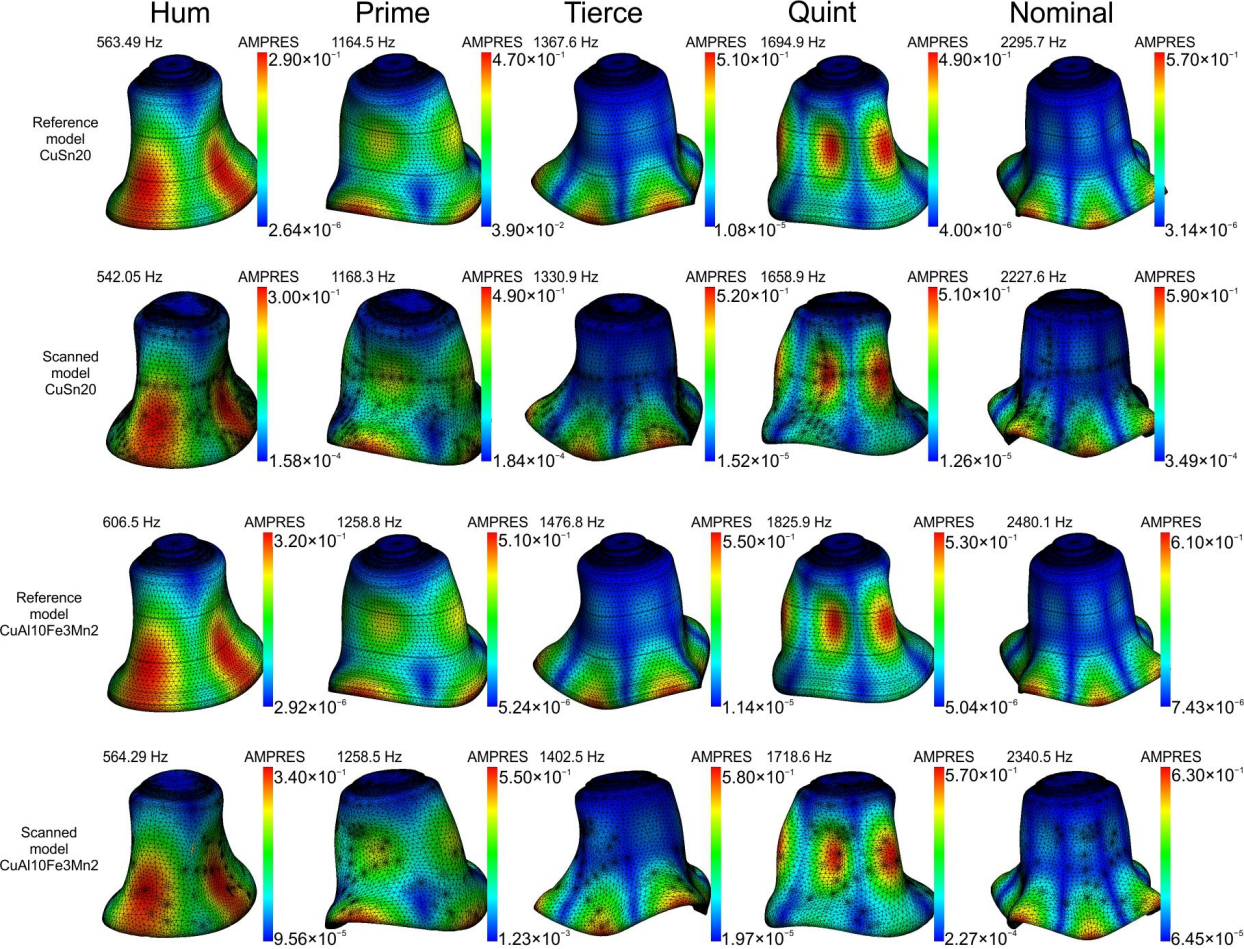
Modal shapes for the musical partials of an analyzed bell predicted by FEM.

The obtained vibration modes correspond to the components of the sound [47,48] (Figure 10).

Numerical and experimental vibration frequencies are summarized in Table 4. Information on the masses of bells (numerical models) and the degrees of freedom of computational models is also presented.

The obtained results indicate discrepancies in the mass and the sound components. At the stage of model comparison, differences between the reference model and in the scanned ones are already noticeable. In the case of bell bronze, the masses differ slightly, while the natural frequencies vary from 0.3% (prime) to about 4% (hum). For aluminum bronze, the mass difference is over 2.5 kg, and the maximum disproportion in frequency is approximately 7.5% (hum).

### 3.2. Experimental Research

**Figure 11:**
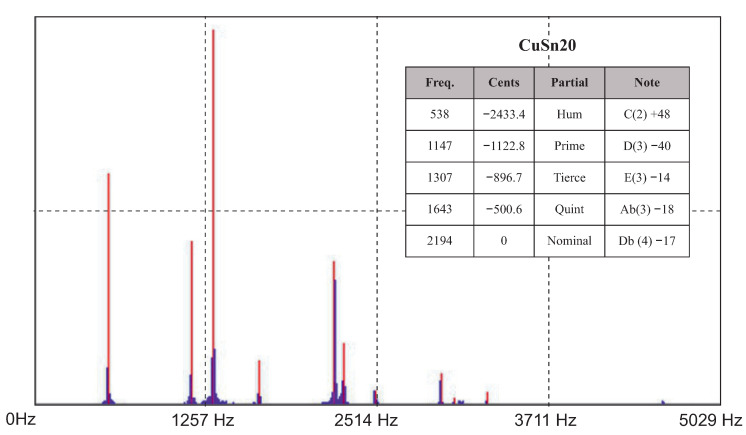
Results from the Wavanal program—bell bronze.

**Figure 12:**
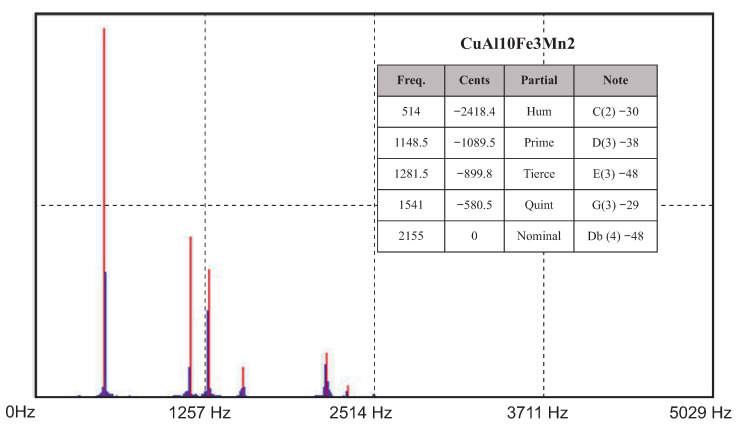
Results from the Wavanal program—aluminum bronze.

Two experimental measurement methods were used to determine the acoustic properties (natural frequencies) of the cast bells. The first measurement was conducted with the help of a voice recorder and the Wavanal program. This free software is used primarily during the bell tuning process, ensuring the possibility of the graphical display of recorded sounds. It allows the identification of frequency components, cents, sound components (partials) and notes. The sound was recorded on two devices and transferred to the software. The graphs (Figure 11 and Figure 12) show the Fourier transforms (on the vertical axis—transform amplitude; on the horizontal axis—frequency) of the recorded sounds. Frequencies determined as partials are highlighted in red. Partials were the same for each analyzed case.

**Figure 13:**
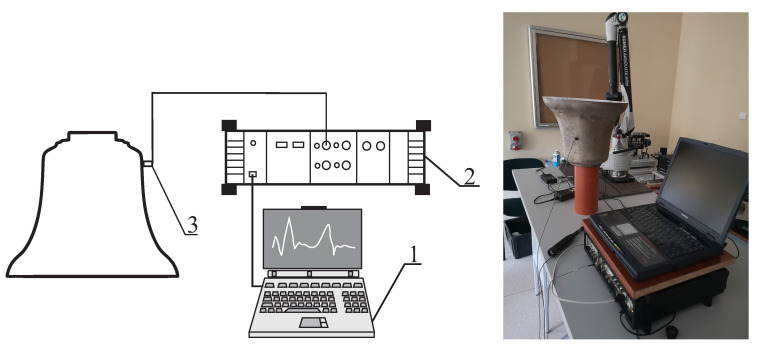
Measuring system for the experimental frequency analysis.

The second measurements were performed using a test stand (Figure 13) equipped with a PC with PULSE Version 7.0—Brüel and Kjaer (1) software, a four-channel vibration analyzer type 3560C (2) and a DeltaTron accelerometer type 4508 B (3).

**Figure 14:**
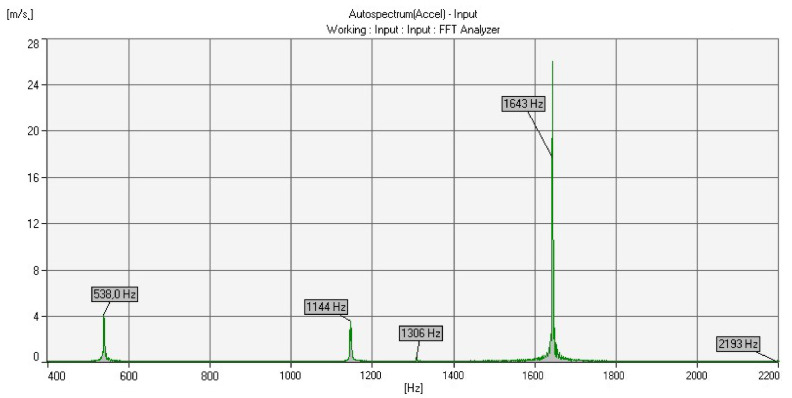
Experimental frequency analysis results—bell bronze.

**Figure 15:**
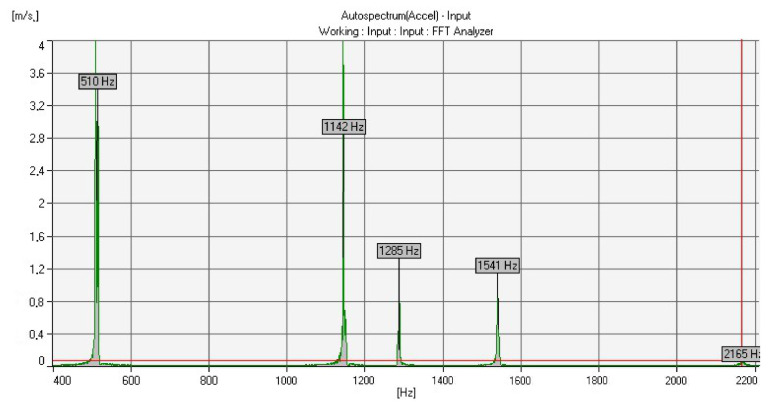
Experimental frequency analysis results—aluminum bronze.

Based on the experimental frequency analysis, the five natural frequencies were obtained, which correspond to the five components of the bell sound (Figure 14 and Figure 15). Table 5 summarizes the vibration frequencies obtained from the experiments (the presented results are the average values from five trials each).

**Figure 16:**
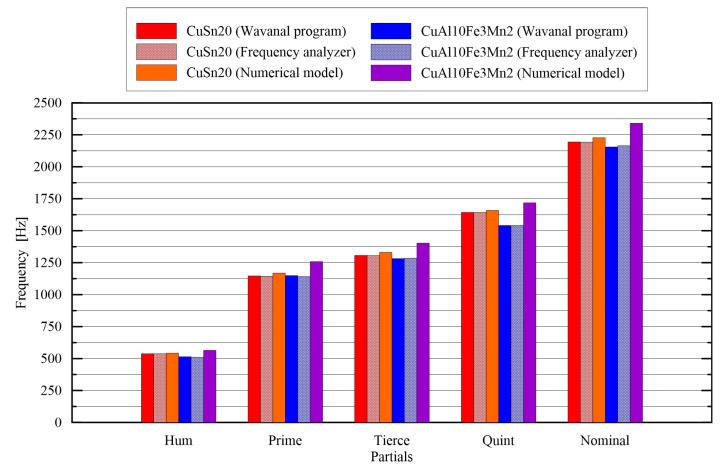
Comparison of the experimental and numerical frequencies.

On the basis of the results (Figure 16), it can be concluded that the vibration frequencies obtained from the Wavanal program and the experimental frequency analysis are almost identical, and the relative error does not exceed 0.8%. Therefore, it can be stated that the experimental tests were performed correctly. Additionally, it can be suggested that free software can be used to determine the acoustic properties of bells without the need for advanced measuring equipment. Examples of other free programs used to analyze these kinds of phenomena are [49]: Rounds (a program for turning recordings of real bells rung singly), Tuner (a spectrum analyzer specifically designed to help with the tuning of bells) and Pitcher (a simple utility for checking the pitch of bell sounds).

The relative error (between the scanned numerical model and cast object) for bell bronze did not exceed 2.15% (prime), and for aluminum bronze 11.6% (quint). The calculations took into account the mechanical properties of CuAl10Fe3Mn2 bronze and not aluminum oxide, which is present in a significant amount in the entire volume of the cast body, which of course affects the obtained results. However, by changing the technology, the formation of aluminum oxide during melting and mold filling can be reduced. In the case of sand casting, Al2O3 occurs in large clusters, crystallizing at the grain boundaries, causing intercrystalline cracks during solidification. It is caused by the difference in the Al2O3 thermal expansion coefficient compared to the base material of the casting. Due to the comparative nature of the tests, extra care was taken not to introduce additional variables which could have influenced on the final sound assessment—keeping the melting technology and the casting mold. Therefore, one cannot only talk about errors in numerical simulations, but must mention not taking into account many phenomena that occur in reality and are not introduced in the virtual models.

## 4. Conclusions

This paper presents a complete analysis of the quality of manufactured bells. Bells made of two materials, CuSn20 and CuAl10Fe3Mn2, with similar acoustic properties were analyzed. The quality analysis was divided into two stages. The first part was the scanning of the bells with the use of the measuring arm with the scanning head. The obtained scans were analyzed for deviations. In the next phase, the Body Compare tool in the SolidWorks program was used to compare the scans with the original design. The maximum differences in thickness and shape were found. The correctness of the adopted foundry shrinkage was also determined. In the second part of the work, the sound quality of the manufactured bells was assessed. Numerical analysis and two experimental studies were performed using the vibration analyzer sensor and the free Wavanal software. The five characteristic bell frequencies were obtained.

Considering the tests, it can be unequivocally stated that the bell cast made of aluminum bronze showed significant deviations in terms of shape and acoustic properties. Assuming that the entire technological process has been performed correctly, the use of this material is not recommended in the bell casting process. When the bronze bell is considered, one can conclude that the obtained results are satisfactory. The existing differences between the numerical model and the cast bell resulted from the porosity of the material, the foundry shrinkage of the casting and the material data adopted in theoretical considerations. In cases of such objects, the appropriate shrinkage should be taken into account when preparing a model. Differences in the dimensions of the object were observed for all scans. Both castings shrunk significantly in the vertical direction (the resulting castings were shorter). Much attention should be paid to the process of placing the protective cover, as in both cases there was a material change on one side. This proves that the distribution of the protective coating was uneven.

At the present stage, the obtained results are sufficient to develop a modified template that will take into account material shrinkage, will eliminate some technological errors and will provide a cast similar to the model. This, in turn, will allow one to obtain the required acoustic properties without additional tuning of the bell.

## Figures and Tables

**Table 1:** Mechanical properties of the alloys used.

	CuSn20	CuAl10Fe3Mn2
**Density** (kg/m${}^{3}$)	8640	7530
**Elastic modulus** (GPa)	117	110
**Poisson ratio**	0.34	0.32
**Elongation** (%)	4	12
**Hardness** (HV)	136	125

**Table 2:** Specifications of scanning sensor (RS3) [35].

**Max. Point Acquisition Rate**	460,000 Points/s	**Points per Line**	4600
**Line rate**	100 Hz	**Stand off**	150 mm ± 50 mm
**Accuracy**	2 sigma/30 μm	**Weight**	340 g
**Line width range** (min/mid/max)	46 mm/65 mm/85 mm	**Min. point spacing**	0.014 mm

**Table 3:** Theoretical intervals for the lowest five partials.

Partial	Interval	Cents
Hum	Two octave below nominal	−2400
Prime	One octave below nominal	−1200
Tierce	Minor third above prime	−900
Quint	Perfect fifth above prime	−500
Nominal	–	0

**Table 4:** Numerical partial frequencies.

	CuSn20		CuAl10Fe3Mn2	
	Reference Model	Scanned Model	Reference Model	Scanned Model
Mass [kg]	28.63	27.40	24.49	21.74
No. of DOF	620,853	1,124,301	620,853	716,724
Partial	[Hz]	[Hz]	[Hz]	[Hz]
Hum	563.49	542.05	606.5	564.29
Prime	1164.5	1168.3	1258.8	1258.5
Tierce	1367.6	1330.9	1476.8	1402.5
Quint	1694.9	1658.9	1825.9	1718.6
Nominal	2295.7	2227.6	2480.1	2340.5

**Table 5:** Experimental partial frequencies.

Partial	CuSn20		CuAl10Fe3Mn2	
	Wavanal Program [Hz]	Frequency Analyzer [Hz]	Wavanal Program [Hz]	Frequency Analyzer [Hz]
Hum	538.0	538.0	514.0	510.0
Prime	1147.0	1144.0	1148.5	1142
Tierce	1307.0	1306.0	1281.5	1285.0
Quint	1643.0	1643.0	1541.0	1541.0
Nominal	2194.0	2193.0	2155.0	2165.0
